# Association between Life’s Essential 8 and estimated pulse wave velocity among adults in the US: a cross-sectional study of NHANES 2011–2018

**DOI:** 10.3389/fpubh.2024.1388424

**Published:** 2024-05-30

**Authors:** Yang Fan, Sisi Yang, Lei Ruan, Cuntai Zhang, Ming Cao

**Affiliations:** Department of Gerontology, Tongji Hospital, Tongji Medical College, Huazhong University of Science and Technology, Wuhan, China

**Keywords:** NHANES, Life’s Essential 8, ePWV, cardiovascular health, vascular aging

## Abstract

**Background:**

Arterial stiffness, typically evaluated via estimated pulse wave velocity (ePWV), is believed to have a significant association with cardiovascular diseases. The objective of this study was to investigate the correlation between Life’s Essential 8 (LE8), a newly revised metric of cardiovascular health, and ePWV among adult population in the United States.

**Methods:**

This research employed a cross-sectional methodology, drawing upon data from the National Health and Nutrition Examination Survey (NHANES) spanning from 2011 to 2018. To explore the relationship between LE8 and ePWV among adults in the US, both univariate and multivariate linear regression analyses were carried out. Additionally, the restricted cubic splines method was utilized to examine any non-linear correlation.

**Results:**

The study comprised 6,742 participants with an average age of 48.30 ± 0.35 years. Among these, 3,236 were males, representing a weighted percentage of 48%. The population’s weighted average LE8 score was 68.68 ± 0.37, while the average ePWV was 8.18 ± 0.04. An entirely adjusted model revealed a negative correlation between ePWV and LE8 scores [in the moderate LE8 group, coefficient − 0.17, 95% CI -0.28 to −0.06, *p* = 0.004; in the high LE8 group, coefficient − 0.44, 95% CI -0.56 to −0.32, *p* < 0.0001]. This negative correlation was consistent with the findings in demographic subgroup analysis, with the effect size being more pronounced among adults under the age of 60, and individuals without hypertension, cardiovascular disease, or diabetes.

**Conclusion:**

Our study reveals a negative correlation between LE8 and ePWV in the adult population of the US, suggesting that LE8 could potentially serve as an indicative marker for evaluating the risk of vascular stiffness. This inverse relationship is markedly stronger in adults below 60 years and those without diagnosed vascular diseases. This implies that lifestyle upgrades and risk factor management could be especially advantageous in curbing arterial stiffness within these groups. These conclusions underscore the importance of primary prevention in mitigating the risk of vascular aging in a relatively healthy group, emphasizing the significance of early intervention and risk factor management in cardiovascular disease.

## 1 Introduction

The Framingham Heart Study, initiated in 1961, highlighted the importance of “risk factors” in cardiology, setting the stage for the development of risk prediction models for cardiovascular diseases (CVD) (1). Building on this foundation, the American Heart Association (AHA) introduced “Life’s Simple 7” (LS7) in 2010 to improve CVH by focusing on cholesterol, blood pressure, glucose levels, smoking status, physical activity, diet, and body mass index (BMI) (2). In a notable advancement, the AHA updated this framework to “Life’s Essential 8” (LE8) last year, enhancing the original metrics and adding sleep health as a critical component of CVH (3). This update reflects a comprehensive approach to CVH, acknowledging the role of lifestyle factors and sleep in influencing cardiovascular outcomes. Recent studies have shown that higher LE8 scores are associated with increased life expectancy among US adults, underscoring the scoring system’s utility in capturing individual differences and the broader determinants of health (4–6). Furthermore, evidence suggests that enhancing CVH in individuals with initially low scores can markedly reduce the incidence of both subclinical and manifest CVD (7–10).

Arterial stiffness represents an early, modifiable phase of CVD with its own predictive value for the disease, often measured by estimated pulse wave velocity (ePWV). While the link between several cardiovascular risk factors (e.g., age, hypertension, diabetes, hyperlipidemia, obesity, smoking) and arterial stiffness has been documented (11–13), the cumulative impact of these factors remains to be fully understood. The synergistic effects of these risk factors, when concurrent, may far exceed the sum of their individual impacts, introducing complexities and significant implications for health. Overlooking this cumulative effect could lead to an underestimation of an individual’s risk of developing CVD, potentially resulting in inadequate clinical interventions and preventative strategies.

Accordingly, this research aims to explore the correlation between LE8 scores and ePWV among adults in the US, to shed light on the cumulative effects of cardiovascular risk factors on arterial stiffness. Through this analysis, we seek to bridge the gap in current understanding regarding how a holistic assessment of CVH may mirror the early and modifiable phases of CVD risk, particularly with respect to arterial stiffness. This study not only meets the demand for a deeper comprehension of the aggregated impacts of these cardiovascular risk factors but also strives to equip medical professionals with actionable insights and strategies for the early detection and intervention of arterial stiffness, thus facilitating the management of individuals at elevated risk for CVD, thereby contributing to the enhancement of cardiovascular health and the prevention of cardiovascular diseases.

## 2 Materials and methods

### 2.1 Study population

This research utilized a population-based, national cross-sectional approach through the National Health and Nutrition Examination Survey (NHANES) database. The NHANES protocol received approval from the Ethics Review Board of the National Centre for Health Statistics, and all participants provided written informed consent. The data, along with analytical method guidelines, are publicly accessible via the US Center for Disease Control and Prevention’s National Center for Health Statistics website at https://www.cdc.gov/nchs/nhanes/index.htm.

**Figure 1:**
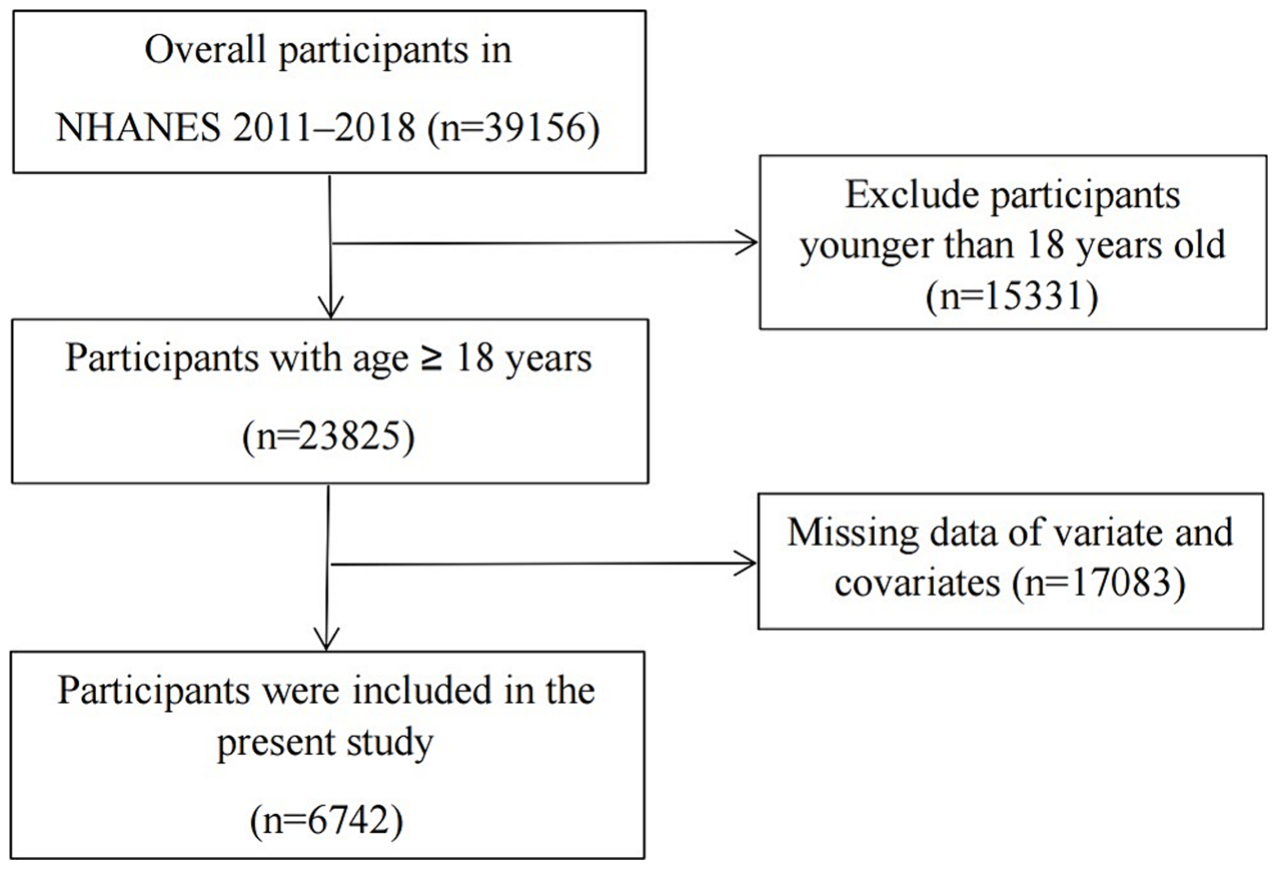
Overview of participants screening.

For this analysis, 39,156 individuals were initially considered from the years 2011 to 2018. Following the application of screening criteria, 6,742 participants were selected to investigate the link between LE8 and ePWV. The process of participant inclusion and exclusion is depicted in Figure 1, with exclusions made for individuals under 18 years of age (*n* = 15,331) and those with incomplete data on LE8, ePWV, or other relevant variables (*n* = 17,083).

### 2.2 Assessment of LE8

The LE8 framework encompasses several components: diet, physical activity, nicotine exposure (including smoking cessation), sleep health, BMI, blood lipids, glucose levels, and blood pressure (3). It adopts a novel scoring system, where each component is rated on a scale from 0 to 100, culminating in a composite cardiovascular health (CVH) score within the same range. Based on the total score, CVH status is classified as poor (<50), moderate (50–79), or good (≥80) (4). This study applies these thresholds to delve into the connection between LE8 components and ePWV.

Dietary scores were determined using average intake data from two 24-h dietary recalls conducted at the study’s outset. Data on physical activity, nicotine use, sleep patterns, and medication usage were gathered through standardized surveys. Dietary scores were graded based on the degree of adherence of the DASH style dietary pattern, and exercise was scored based on the number of minutes of moderate-to-high physical activity per week. Nicotine exposure included traditional smoking or exposure to secondhand smoke, and sleep health was scored according to the average sleep time per night. Body measurements for calculating BMI were taken, with BMI itself determined by dividing weight in kilograms by the square of height in meters. Furthermore, NHANES protocols were followed for measuring fasting blood sugar (FBS) and blood pressure, and blood samples were obtained to assess lipid levels and FBS.

### 2.3 Assessment of ePWV

The ePWV value was derived from the participant’s age and mean blood pressure (MBP) utilizing a specific equation (14):



$$\begin{array}{l} ePWV=9.587-0.402\times age+4.560\times 10^{-3}\times age^{2}-\\ 2.621\times 10^{-5}\times age^{2}\times MBP+3.176\times 10^{-3}\times \\ age\times MBP-1.832\times 10^{-2}\times MBP \end{array}$$

The MBP itself was computed from diastolic blood pressure (DBP) and systolic blood pressure (SBP) based on a predetermined formula (15):



MBP=DBP+0.4(SBP−DBP)

All personnel performing BP measurements were formally trained to follow standardized BP measurement guidelines using validated BP measurement equipment to ensure consistency and accuracy of the measurements. Following the calculation of bilateral ePWV values, the higher value was selected for further examination.

### 2.4 Assessment of covariates

Information on age, sex, ethnicity, educational attainment, and family income was collected through self-reporting. Ethnicity was categorized as non-Hispanic white, non-Hispanic black, Mexican American, or other. Education was classified as college or above, high school or equivalent, and less than high school. The presence or absence of systemic diseases, such as diabetes mellitus (DM), including impaired fasting glucose (IFG) and impaired glucose tolerance (IGT), hypertension, and cardiovascular disease, were recorded as binary responses, indicated by a simple “Yes/No.”

### 2.5 Statistical analysis

For continuous variables, weighted means and standard errors were reported, while categorical variables were expressed in terms of frequency and percentage. The ANOVA test or Chi-squared test were applied to compare continuous or categorical variables across different LE8 groups, respectively. For the univariable analysis of covariates, a univariable model was utilized.

The association between LE8 and ePWV was evaluated using survey-weighted linear regression to identify the β-coefficient and 95% confidence interval (CI). Three models were deployed: an unadjusted crude model, Model 1 (adjusted for age and sex), Model 2 (adjusted for age, sex, ethnicity, education, and poverty), and Model 3 (fully adjusted for all covariates, including age, sex, ethnicity, education, poverty, and clinical conditions such as coronary heart disease and diabetes mellitus).

CVH scores were used to divide LE8 values into three subgroups, with the low LE8 group serving as the reference. R software version 4.2.2 was used for all analyses, with a two-sided *p*-value of less than 0.05 considered statistically significant.

## 3 Results

### 3.1 Baseline characteristics

The study sample comprised 6,742 participants aged 18 or above, drawn from the years 2011–2018. The baseline characteristics of the study population, divided by LE8 levels according to their scores, are presented in Table 1. The mean age (standard deviation, SD) of the participants was 48.30 ± 0.35 years, with males making up 48% of the participants (weighted percentage = 3,236). The population had a mean LE8 score (SD) of 68.68 ± 0.37 and a mean ePWV (SD) of 8.18 ± 0.04.

**Table 1:** Baseline characteristics and ePWV of the overall population and stratified by LE8 scores in the NHANES 2011–2018.

Characteristics	Overall	LE8			*p* value
		Low < 50	Moderate (50,80)	High ≥ 80	
Number	6,742	873	4,428	1,441	
LE8	68.68 (0.37)	42.09 (0.27)	66.14 (0.17)	86.94 (0.19)	< 0.0001
ePWV	8.18 (0.04)	9.22 (0.08)	8.39 (0.05)	7.20 (0.06)	< 0.0001
Age	48.30 (0.35)	55.06 (0.63)	49.63 (0.43)	41.83 (0.57)	< 0.0001
Poverty	2.98 (0.06)	2.22 (0.09)	2.93 (0.06)	3.41 (0.07)	< 0.0001
Sex					< 0.0001
Male	3,236 (48)	411 (45.96)	2,247 (50.63)	578 (41.16)	
Female	3,506 (52)	462 (54.04)	2,181 (49.37)	863 (58.84)	
Race/Ethnicity					< 0.0001
Non-Hispanic White	2,789 (41.37)	371 (65.61)	1839 (67.85)	579 (68.61)	
Non-Hispanic Black	1,418 (21.03)	245 (15.74)	991 (11.65)	182 (6.69)	
Mexican American	870 (12.9)	106 (7.79)	583 (7.75)	181 (7.94)	
Other Races	1,665 (24.7)	151 (10.87)	1,015 (12.75)	499 (16.76)	
Education level					< 0.0001
College or above	3,967 (58.84)	361 (43.22)	2,510 (62.37)	1,096 (80.43)	
High School or equivalent	1,475 (21.88)	247 (33.16)	1,027 (23.64)	201 (13.46)	
Less than high school	1,300 (19.28)	265 (23.62)	891 (13.99)	144 (6.11)	
Cardiovascular disease					< 0.0001
No	5,974 (88.61)	649 (76.70)	3,928 (90.39)	1,397 (97.34)	
Yes	768 (11.39)	224 (23.30)	500 (9.61)	44 (2.66)	
Diabetes mellitus					< 0.0001
No	4,106 (60.9)	247 (31.78)	2,600 (63.49)	1,259 (89.50)	
IFG	689 (10.22)	111 (15.15)	509 (12.61)	69 (4.42)	
IGT	478 (7.09)	56 (6.51)	343 (6.91)	79 (4.34)	
DM	1,469 (21.79)	459 (46.57)	976 (16.99)	34 (1.73)	
Hypertension					< 0.0001
No	5,478 (81.25)	515 (62.29)	3,555 (82.66)	1,408 (98.50)	
Yes	1,264 (18.75)	358 (37.71)	873 (17.34)	33 (1.50)	

Compared to those with low LE8 scores, individuals with moderate or high LE8 scores were typically younger, more likely to be female, wealthier, more educated, and had a lower prevalence of cardiovascular disease, diabetes mellitus, and hypertension. Moreover, those with moderate or high LE8 scores exhibited lower ePWV.

### 3.2 Association between LE8 scores and ePWV

The multiple univariable analysis displayed in Table 2 revealed significant associations between ePWV and various factors, including age, poverty, ethnicity, educational attainment, and LE8 score. The relationship between LE8 scores and ePWV was further investigated using survey-weighted linear regression.

**Table 2:** Risk factors for ePWV in adults in NHANES 2011–2018.

Variable	β (95% CI)	*p* value
LE8	−0.05 (−0.05, −0.04)	<0.0001
LE8_group		
Low	0 (reference)	
Moderate	−0.83 (−1.02, −0.64)	<0.0001
High	−2.01 (−2.19, −1.84)	<0.0001
Age	0.11 (0.11, 0.11)	<0.0001
Sex		
Male	0 (reference)	
Female	−0.06 (−0.16, 0.05)	0.29
Poverty	0.09 (0.04, 0.13)	<0.001
Race/Ethnicity		
Non-Hispanic White	0 (reference)	
Non-Hispanic Black	−0.33 (−0.49, −0.17)	<0.0001
Mexican American	−0.99 (−1.18, −0.80)	<0.0001
Other Races	−0.75 (−0.91, −0.59)	<0.0001
Education level		
College or above	0 (reference)	
High School or equivalent	0.33(0.13, 0.53)	0.002
Less than high school	0.37(0.19, 0.55)	<0.001
Cardiovascular disease		
No	0 (reference)	
Yes	2.23(2.05, 2.42)	<0.0001
Diabetes mellitus		
No	0 (reference)	
IFG	1.26 (1.06, 1.45)	<0.0001
IGT	1.15 (0.89, 1.41)	<0.0001
DM	1.91 (1.74, 2.08)	<0.0001
Hypertension		
No	0 (reference)	
Yes	2.79 (2.64, 2.94)	<0.0001

In the unadjusted model, participants with high LE8 levels had significantly lower ePWV (β, −0.83; 95% CI -1.02, −0.64; *p* < 0.0001) compared to those with moderate (β, −2.01; 95% CI, −2.19, −1.84, *p* < 0.0001) and low LE8 levels, as demonstrated by the scatter plot presented in the Supplementary materials, a negative association is observed between the LE8 scores and ePWV (Supplementary Figure S1). This trend persisted even after controlling for age and sex in Model 1, and additional adjustments for ethnicity, education, and poverty in Model 2, as well as comorbidities like cardiovascular disease and diabetes mellitus in Model 3.

Although the association was somewhat weakened after multivariable adjustment in Model 3, individuals with moderate and high LE8 scores still displayed a reduced ePWV (β, −0.17; 95% CI −0.28, −0.06; *p* < 0.0001 and β, −0.44; 95% CI −0.56, −0.32, *p* < 0.0001, respectively) compared to those in the low LE8 group. The P for trend was less than 0.0001 across all models (Table 3).

**Table 3:** Survey-weighted association between LE8 with ePWV in the study population.

Variable	β (95%CI)							
	Crude model		Model 1		Model 2		Model 3	
LE8	β (95%CI)	*p* value	β (95%CI)	*p* value	β (95%CI)	*p* value	β (95%CI)	*p* value
Low	0 (reference)		0 (reference)		0 (reference)		0 (reference)	
Moderate	−0.83 (−1.02, −0.64)	<0.0001	−0.27 (−0.37, −0.17)	<0.0001	−0.22 (−0.32, −0.12)	<0.0001	−0.17 (−0.28, −0.06)	0.004
High	−2.01 (−2.19, −1.84)	<0.0001	−0.61 (−0.72, −0.51)	<0.0001	−0.52 (−0.62, −0.41)	<0.0001	−0.44 (−0.56, −0.32)	<0.0001
p for trend		<0.0001		<0.0001		<0.0001		<0.0001

Crude model: adjusted for none. Model 1: adjusted for age and sex. Model 2: adjusted for age, sex, ethnicity, education level and poverty. Model 3: adjusted for age, sex, ethnicity, education level, poverty, history of cardiovascular disease and diabetes mellitus.

**Figure 2:**
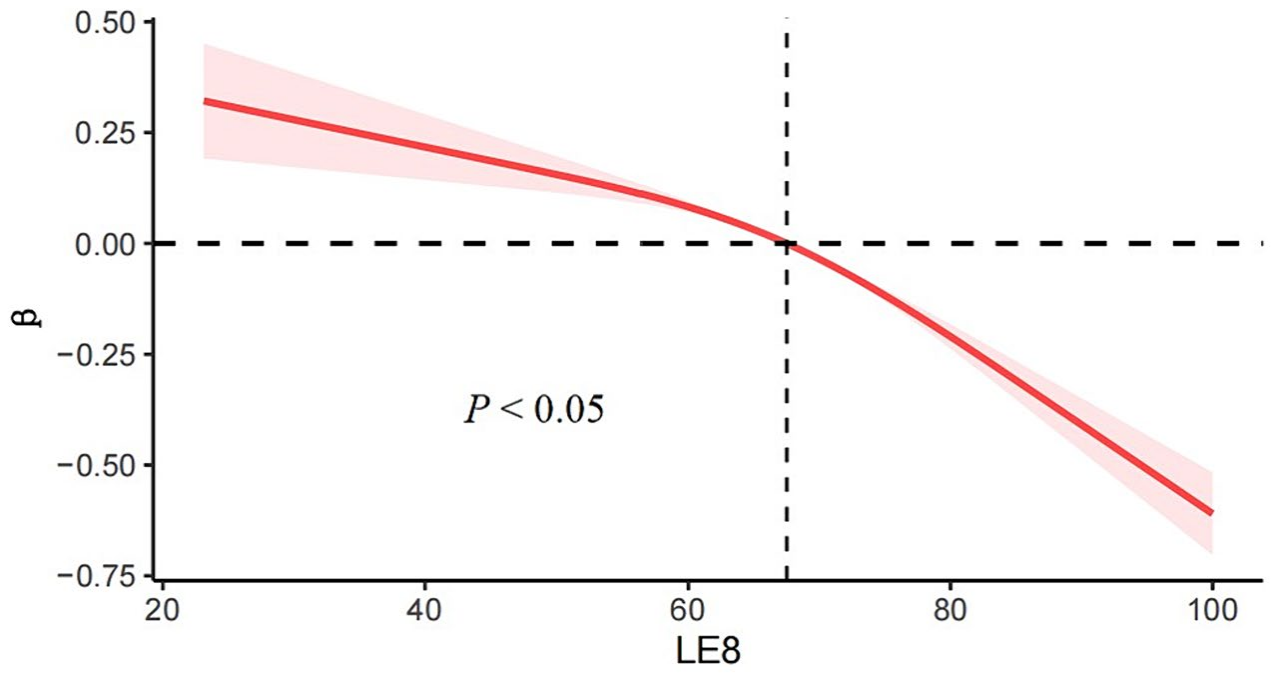
RCS (Restricted Cubic Splines) curve analysis predicting relationship between LE8 and ePWV.

Based on the RCS (Restricted Cubic Splines) curve analysis provided in Figure 2, the decreasing linear relationship between LE8 and ePWV was seen, demonstrated that with an increase in LE8, there is a tendency for PWV to decrease, suggesting that higher LE8 correlates with lower arterial stiffness and potentially better vascular health. The LE8 and ePWV association exhibits significant nonlinear relationship (*p* < 0.05), particularly among individuals with higher LE8 (score > 67.5), further supporting the critical role of lifestyle improvements and health risk factor management for primary prevention in reducing vascular risk.

### 3.3 Subgroup analyses

Table 4 presents the subgroup analyses, which suggest variations in the association between LE8 scores and ePWV across different age categories (< 60 years versus ≥60 years) and genders, with the P for trend being <0.0001, =0.016, <0.0001, and < 0.0001, respectively. Notably, the influence of LE8 scores on ePWV was more noticeable in participants aged under 60 years.

**Table 4:** Subgroups analyses.

Subgroups	LE8					P for trend
	Low	Moderate	*p* value	High	*p* value	
Age						
<60	0 (reference)	−0.802 (−0.958, −0.646)	<0.0001	−1.547 (−1.714, -1.379)	<0.0001	<0.0001
> = 60	0 (reference)	0.133 (−0.129, 0.394)	0.314	−0.44 (−0.757, −0.123)	0.007	0.016
Sex						
Male	0 (reference)	−1.073 (−1.293, −0.852)	<0.0001	−2.013 (−2.241, −1.785)	<0.0001	<0.0001
Female	0 (reference)	−0.678 (−0.951, −0.405)	<0.0001	−2.054 (−2.310, −1.798)	<0.0001	<0.0001
HBP						
No	0 (reference)	−0.559 (−0.725, −0.392)	<0.0001	−1.402 (−1.604, −1.200)	<0.0001	<0.0001
Yes	0 (reference)	0.027 (−0.236, 0.290)	0.837	1.204 (0.374, 2.035)	0.005	0.166
CVD						
No	0 (reference)	−0.852 (−1.051, −0.653)	<0.0001	−1.913 (−2.087, −1.739)	<0.0001	<0.0001
Yes	0 (reference)	0.192 (−0.201, 0.586)	0.331	−0.628 (−1.222, −0.033)	0.039	0.58
DM						
No	0 (reference)	−0.735 (−1.047, −0.422)	<0.0001	−1.638 (−1.907, −1.369)	<0.0001	<0.0001
IFG	0 (reference)	−0.225 (−0.692, 0.242)	0.338	−0.794 (−1.529,−0.059)	0.035	0.047
IGT	0 (reference)	0.023 (−0.648, 0.694)	0.945	0.214 (−0.550, 0.978)	0.574	0.512
DM	0 (reference)	−0.132(−0.422, 0.158)	0.365	−1.007 (−1.777, −0.237)	0.011	0.102

Adjusted for ethnicity, education level and poverty. HBP, hypertension; CVD, cardiovascular disease; DM, diabetes millitus.

In addition, when conducting a subgroup analysis based on the history of hypertension, cardiovascular disease (CVD), and metabolic diseases (MD), there were significant inverse relationships between LE8 scores and ePWV, particularly with a strong association observed in adults who did not have these conditions (all P for trend <0.0001).

## 4 Discussion

Our study examined a nationally representative cohort of US adults and discovered a significant association between the recently devised LE8 metric, a measure of cardiovascular health (CVH), and estimated pulse wave velocity (ePWV). We found an inverse correlation between LE8 and ePWV, suggesting that LE8 could serve as a helpful tool for understanding ePWV and, by extension, arterial stiffness.

At present, ePWV is the most extensively researched index of arterial stiffness and satisfies most of the strict criteria required for a clinically relevant biomarker (16). Such research can lead to new theories about vascular aging that can be evaluated in meticulously planned prospective studies using more sophisticated vascular measures (17). This implies that ePWV could be employed to assess blood pressure in large-scale epidemiological cohort studies. To our knowledge, this is the first study to explore the relationship between the newly introduced LE8 metric and ePWV in US adults.

Recognized cardiovascular risk factors such as hypertension, diabetes mellitus, hyperlipidemia, obesity, and smoking have been linked to arterial stiffness (18, 19). We further investigated the cumulative impact of these risk factors on the progression of arterial stiffness. In our research, we observed a negative linear relationship between ePWV and LE8 scores, which persisted even after accounting for age, sex, race, education level, and history of hypertension, cardiovascular disease (CVD), and metabolic diseases (MD). Higher levels of CVH may help reduce CVD-related morbidity and mortality, possibly by slowing down the process of arterial stiffness. To our knowledge, several studies have investigated the relationship between LE8 and life expectancy.

Arterial stiffness is an early and reversible stage of CVD and an important link in the chain of cardiovascular events. Cardiovascular risk factors (e.g., smoking, hypertension, diabetes mellitus, and hypercholesterolemia) independently predict CVD development (20). In addition to strengthening the targeting of traditional cardiovascular risk factors, studies have shown that lifestyle improvements (e.g., aerobic exercise, weight loss, sodium restriction, and other preventive measures targeting risk factors) can help slow the progression of arterial stiffness, thereby effectively reducing the incidence of CVD.

Previous research has indicated that diabetes and metabolic shifts predict arterial stiffness in a dose-responsive manner (21). Chronic hyperglycemia and hyperinsulinemia enhance the activity of the local renin-angiotensin-aldosterone system, modifying the mechanical attributes of the arterial wall tissue. Persistent elevation of mean blood pressure also results in arterial wall thickening, primarily in the middle layer. A J-shaped correlation exists between alcohol consumption and ePWV, and among diet-related factors, salt intake may have the most substantial effect on vascular stiffness (22). Current dietary levels of salt supplementation lead to a dose-dependent rise in blood pressure (23). One study noted that salt-sensitive rats on a high-salt diet displayed increased vascular stiffness and altered arterial wall composition weeks before blood pressure increased (24). These lifestyle and risk factors eventually result in changes in endothelial function, smooth muscle tone, vascular intima-media thickness, and the composition of collagen, fibronectin, hyaluronic acid, and elastin.

Compared to LS7, LE8 incorporates sleep as a factor. A meta-analysis showed that both short and long sleep durations were associated with ePWV compared to the recommended sleep duration (25). In subgroup analyses, meaningful associations were found between short sleep duration and elevated PWV in adults with cardiometabolic diseases, and between long sleep duration and elevated PWV in older adults. These results suggest that both short and long sleep durations may contribute to subclinical CVD. Hence, LE8 offers more value than LS7 in assessing CVH and predicting CVD risk. Moreover, ePWV, which can noninvasively detect arterial stiffness, is convenient, quick, economical, and highly valuable in clinical applications.

Our study’s strengths include the use of nationally representative data, a new LE8 score to examine the relationship between ePWV and life expectancy among adults in the US, and stratified analyses by age, gender, and clinical disease subgroups. This study found a negative relationship between the LE8 score and ePWV, notably among adults under 60 and those without hypertension, cardiovascular disease, or diabetes. This finding provides a further basis for the development of cardiovascular health assessment and preventive measures, and the LE8 score can be used as an indicator to assess the risk of vascular stiffness, helping in the early identification of people at high risk for cardiovascular disease. The study, however, has some limitations. First, lifestyle factors, such as diet and sleep duration, were self-reported in the NHANES, which inevitably leads to measurement errors. Future studies could improve data quality through objective measures (e. g. using wearable devices to monitor sleep and activity levels, or optimizing food questionnaires and validated by professionals). Second, as ePWV information was only available at baseline, potential changes in ePWV during the follow-up period were not considered. Further studies are needed to evaluate the relationship between PWV changes and health outcomes. Third, in the analyses of the LE8 score, each metric closely mirrored the risk factor for arterial stiffness, estimated by ePWV. Fourth, e-cigarette information wasn’t included in the LE8 score calculation, as it was only available post-2013, possibly leading to a slight overestimation of the LE8 score. With the increasing popularity of e-cigarette use, future research should incorporate e-cigarette use in cardiovascular health assessments to more accurately assess the impact of tobacco use on cardiovascular health. Furthermore, future studies are needed to overcome existing limitations through long-term tracking and using objective measures of health indicators, and to explore the relationship of multidimensional data (e. g. genetic information, environmental exposures, etc.) to cardiovascular health that can provide deeper insights. While further exploration of customized interventions based on LE8 scores, such as targeted lifestyle modification recommendations, can verify whether improving LE8 scores can significantly reduce cardiovascular outcomes. Through these research findings and practice improvements, we can better understand and respond to CVD prevention, especially through early intervention to improve the overall public cardiovascular health and lifestyle improvement.

## 5 Conclusion

To summarize, our research findings reveal that in the US adult population, LE8 exhibits a significant and inverse relationship with ePWV. This suggests that enhancing levels of cardiovascular health (CVH) could effectively decelerate the development and progression of vascular aging indicators, notably arterial stiffness, thereby potentially decreasing cardiovascular disease (CVD) prevalence. The application of the CVH framework in the management of clinical vascular health, particularly in the context of primary prevention of vascular aging, warrants strong endorsement.

In conclusion, the present study demonstrated that LE8 is independently and negatively associated with ePWV among adults in the US and that increasing CVH levels has the potential to slow the onset and progression of vascular ageing marker-arterial stiffness, thus reducing the burden of CVD. Rational use of the CVH concept in clinical vascular health management, especially for the primary prevention for vascular ageing should be advocated.

## Data availability statement

Publicly available datasets were analyzed in this study. This data can be found at: https://www.jianguoyun.com/p/DUuVCk8Q64SzDBjD1sMFIAA; https://www.jianguoyun.com/p/Dd4TsZoQ64SzDBjJ1sMFIAA.

## Ethics statement

The studies involving humans were approved by Ethics Reviewed Board of the National Centre for Health Statistics. The studies were conducted in accordance with the local legislation and institutional requirements. The participants provided their written informed consent to participate in this study.

## Author contributions

YF: Formal analysis, Writing – original draft, Writing – review & editing. SY: Data curation, Methodology, Software, Writing – review & editing. LR: Writing – review & editing. CZ: Funding acquisition, Writing – review & editing. MC: Data curation, Formal analysis, Funding acquisition, Methodology, Software, Writing – original draft, Writing – review & editing.
