# Supplementary figures and images for: Association between Life’s Essential 8 and estimated pulse wave velocity among adults in the US: a cross-sectional study of NHANES 2011–2018
Source: Front Public Health. 2024 May 30;12:1388424. doi: 10.3389/fpubh.2024.1388424 (PMC11169870; doi:10.3389/fpubh.2024.1388424)

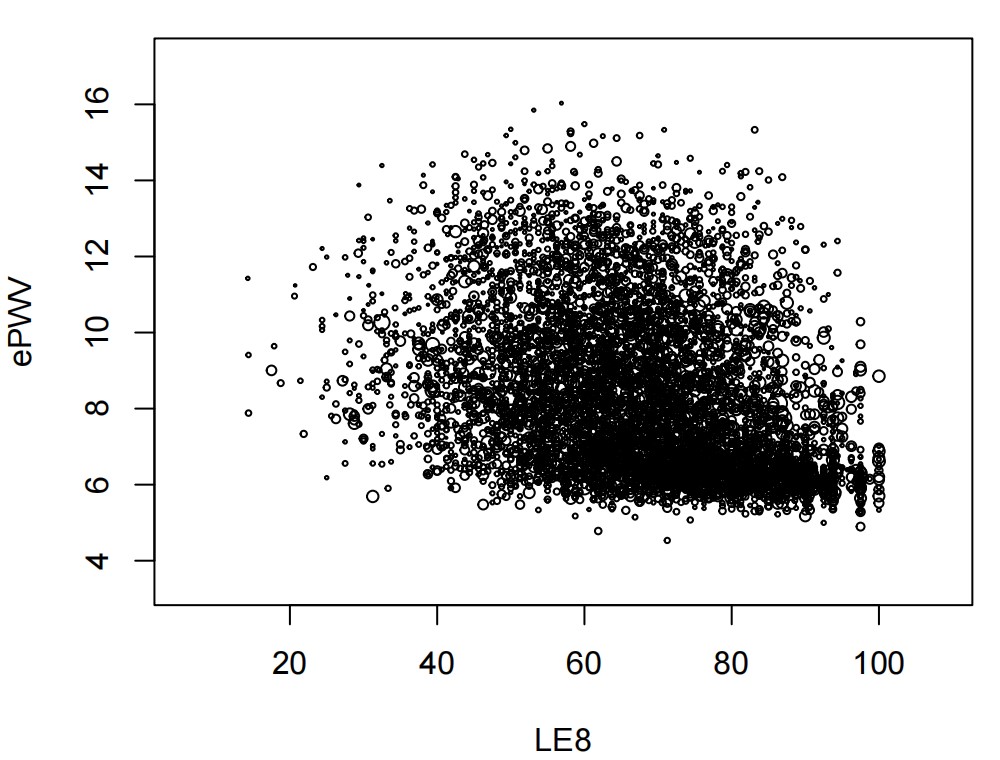

Supplement: Supplementary file 1 [file Image_1.JPEG]
